# Macrophage Function Modulated by tPA Signaling in Mouse Experimental Kidney Disease Models

**DOI:** 10.3390/ijms241311067

**Published:** 2023-07-04

**Authors:** Ling Lin, Kebin Hu

**Affiliations:** 1 Division of Nephrology, Department of Medicine, Penn State University College of Medicine, Hershey, PA 17033, USA; 2 Department of Cellular and Molecular Physiology, Penn State University College of Medicine, Hershey, PA 17033, USA

**Keywords:** tissue plasminogen activator (tPA), macrophage function, signal transduction, inflammation, nuclear factor- κB (NF-κB), low-density lipoprotein receptor-related protein (LRP-1), annexin A2, macrophage polarization, kidney disease

## Abstract

Macrophage infiltration and accumulation is a hallmark of chronic kidney disease. Tissue plasminogen activator (tPA) is a serine protease regulating the homeostasis of blood coagulation, fibrinolysis, and matrix degradation, and has been shown to act as a cytokine to trigger various receptor-mediated intracellular signal pathways, modulating macrophage function in response to kidney injury. In this review, we discuss the current understanding of tPA-modulated macrophage function and underlying signaling mechanisms during kidney fibrosis and inflammation.

## 1. Introduction

Macrophages, as a critical component of innate immunity, play a fundamental role in immune defense against pathogens, as well as in the modulation of inflammatory responses. In general, macrophages within various tissues and organs are differentiated from bone marrow myeloid progenitor-derived monocytes in the circulation and undergo a closely regulated process of adaption to the local microenvironment [1]. However, mounting evidences have shown that macrophages are also derived from embryonic yolk sac and fetal liver and become a part of the local resident cell population participating in organ-specific functions [2].

Macrophages are not homogenous and consist of variably mixed populations in various organs, such as liver Kupffer cells and brain microglial cells, that carry out local microenvironment-specific functions [3]. Under various physiological or pathological conditions, macrophages acquire distinct functional phenotypes through polarization that are generally categorized into two subsets as either classically activated (M1) or alternatively activated (M2). In general, M1 macrophages garner high motility and promote inflammation and damage through a combination of transcription factors such as NF-κB, whereas M2 macrophages help to resolve inflammation and promote tissue remodeling [4]. Of note, M1 and M2 only represent two extremities of a full spectrum of macrophage polarization, and most differentiated macrophages fall into various states of polarization between M1 and M2. Additionally, macrophage polarization is a dynamic process, and phenotypic switch between M1 and M2 happens under various pathological conditions [5].

One of the histological hallmarks of chronic kidney disease (CKD) is renal infiltration and accumulation of inflammatory cells, such as macrophages. Macrophage infiltration and accumulation has been shown to correlate with the severity of kidney damage in human patients with chronic kidney disease [6,7]. While the recruitment of inflammatory cells to the sites of injury is an essential part of the wound healing process, sustained macrophage infiltration in response to chronic injury will eventually become pathological and cause disorganized healing and remodeling, leading to irreversible tissue destruction and organ dysfunction and deterioration. Thus, better understanding of the regulation of macrophage functions, as well as their roles in disease pathogenesis, will help the development of selective and effective therapies.

## 2. tPA Structure and Dual Functions of Protease and Cytokine

Tissue plasminogen activator (tPA), a 69 kDa glycoprotein consisting of 527 or 530 amino acids, is a member of the serine protease family that plays a pivotal role in the homeostasis of blood coagulation and fibrinolysis, as well as matrix regulation [8]. tPA is synthesized within cells and released as a single-chain enzyme, which is subsequently cleaved by plasmin into a two-chain form that consists of a heavy chain and a light chain. The single-chain tPA contains four domains: (i) a finger (F) domain, which is homologous to the first domain of the fibronectin; (ii) an EGF domain, which is homologous to EGF; (iii) two Kringle (K) domains; and (iv) the catalytic protease (P) domain. The heavy chain of the two-chain tPA contains F, EGF, and K domains, while the light chain contains the P domain. The F domain is essential for the binding of tPA to fibrin and interactions with tPA-associated receptors, such as low-density lipoprotein receptor-related protein (LRP-1) and annexin A2. The EGF domain allows tPA to interact with EGF receptors. The Kringle domains contain active sites with an affinity for lysine in mediating protein–protein interaction. The P domain of tPA consists of Histidine 322, Asparagine 371, and Serine 478 [9], which are critical to the protease functions of tPA. The single mutation of these sites, such as Serine 478 to Alanine, renders tPA catalytical inactivation, while its other functions remain intact [10]. This mutant non-enzymatic tPA plays an instrumental role in identifying the protease-independent effects of tPA.

As a serine protease, tPA and its cousin serine protease urokinase plasminogen activator (uPA) cleave plasminogen into biologically active plasmin, which degrades insoluble fibrin fibers and initiates the fibrinolytic process. Additionally, tPA also regulates the degradation of extracellular matrix (ECM) through plasmin-activated matrix metalloproteinases (MMPs) [8,11]. Besides its protease activities, we and others, using the above-mentioned non-enzymatic tPA, have shown that tPA acts as a cytokine triggering multiple signal pathways to regulate various cellular processes [12,13,14,15,16] and is involved in the pathogenesis of numerous disease models, including liver fibrosis, ischemic brain injury, and chronic kidney disease [8,11,17,18].

## 3. Renal Origin and Distribution of tPA after Kidney Injury

tPA is produced and released by vascular endothelial cells in the circulation to maintain blood flow through anticoagulation and fibrinolysis. Within endothelial cells, tPA is stored in Weibel–Palade bodies, which also store von Willebrand factor, an important player in blood coagulation cascade [19]. A variety of stimuli, such as vascular endothelial growth factor, fluid shear stress, and thrombin, induce tPA mRNA expression in endothelial cells [20].

The normal renal expression of tPA mRNA and protein is very low and usually hard to detect [21,22]. However, tPA mRNA is detected by in situ hybridization analysis in glomerular endothelial cells, podocytes, and the epithelial cells of the distal collecting ducts [21]. tPA mRNA expression is induced in the damaged proximal tubules after acute kidney injury (AKI) [21]. In a unilateral ureter obstruction (UUO)-induced CKD model, both tPA mRNA and protein levels are dramatically induced in the renal interstitium [13,14,22]. Interstitial parenchymal cells, such as fibroblasts, as well as infiltrated inflammatory cells in the diseased kidney, such as macrophages, may also contribute to the interstitial induction of tPA [23]. Intriguingly, our previous work has demonstrated that myeloid cells are the primary source of obstructive injury-induced tPA responsible for renal fibrogenesis and inflammation [24]. Specifically, we have generated chimeric mice that lack tPA in either myeloid cells or renal parenchyma by bone marrow transplantation between tPA wildtype (WT) and knockout (KO) mice (bone marrow donor/recipient: WT/WT, WT/KO, KO/WT, and KO/KO) followed by UUO challenge. Notably, it has been found that WT/WT and WT/KO mice (myeloid cells with WT tPA) display similar and dramatically increased renal fibrosis and macrophage accumulation, whereas KO/WT and KO/KO mice (tPA-deficient myeloid cells) show similar but significantly reduced fibrosis and macrophage infiltration. Additionally, tPA in the circulation has little effect on the renal accumulation of macrophages and fibrosis. Thus, myeloid cells are the main source of renal endogenous tPA responsible for obstructive injury-induced macrophage accumulation and renal fibrosis.

## 4. Renal Receptors of tPA Signaling

**Figure 1:**
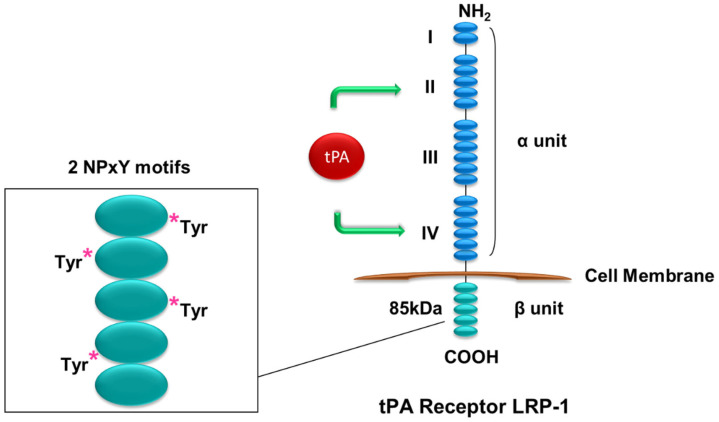
**Structure of LRP-1.** LRP-1 has an extracellular α subunit containing tPA-binding II and IV domains and an intracellular β subunit with two NPxY motifs and several tyrosine residues that can be phosphorylated to initiate signal transduction. * stands for phosphorylation.

tPA does not have a dedicated or specific receptor. However, tPA is known to bind to cell surface LRP-1 and annexin A2. Mature LRP-1 is a 600 kDa transmembrane protein first identified as a tPA receptor in hepatocytes [25]. LRP-1 consists of an extracellular 515 kDa α subunit and an 85 kDa β subunit. The LRP-1 α subunit consists of an extracellular segment comprising domains I, II, II, and IV, while the β subunit contains a transmembrane domain and a cytosolic tail harboring two NPxY motifs and numerous tyrosine residues [26,27,28,29] (Figure 1). tPA has been shown to bind domains II and IV in the α subunit and can induce the phosphorylation of tyrosine site(s) in the β subunit [15,26,30]. Phosphorylation of the tyrosine residue(s) is essential for LRP-1 to relay its signal, of which Tyr4507 phosphorylation mediates tPA-induced renal fibroblast proliferation through activation of its downstream, the extracellular signal-regulated kinase 1/2 (Erk1/2) and the p90 ribosomal S6 kinase (p90RSK) pathway [15]. It remains unknown how exactly the binding of tPA to LRP-1 induces phosphorylation of LRP-1 tyrosine residue(s) and subsequent activation of downstream signal mediators. Tyrosine residues on the β subunit of LRP-1 are known to provide docking sites for signaling adaptor proteins, including Shc [31,32,33], which upon phosphorylation will then recruit Grb2-Sos and activate Ras signaling [33]. Tyr4507 phosphorylation induced by v-Src has been found to cause the association of LRP-1 and Shc [31,34]. Thus, Shc likely mediates tPA/LRP-1-induced Ras-Erk1/2 signaling. tPA and LRP-1 mediates multiple signaling cascades and influences various cellular processes, including ECM remodeling, myofibroblast activation, and fibroblast accumulation and proliferation, as well as macrophage survival and peritoneal macrophage efflux [13,14,15].

**Figure 2:**
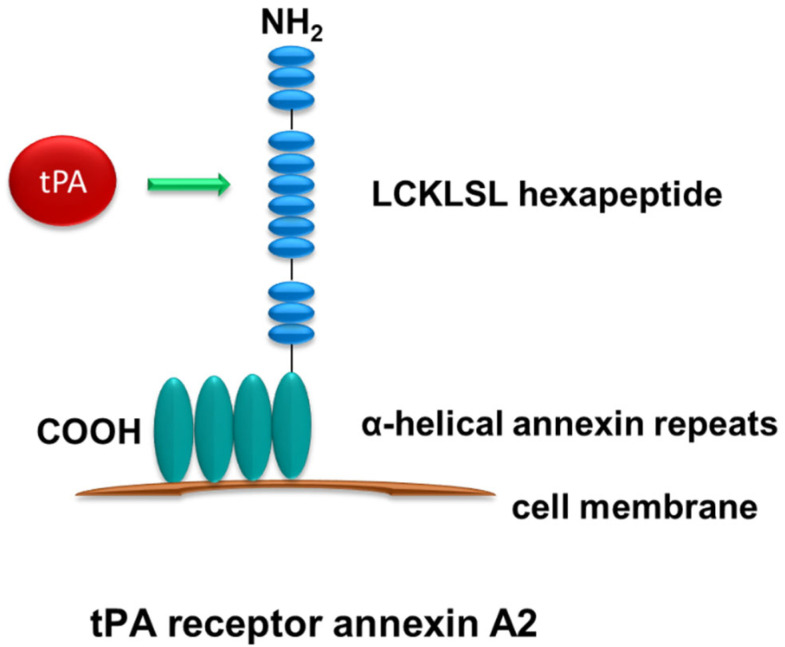
**Structure of annexin A2.** Annexin A2 has no transmembrane domain and docks onto the cell surface through its four highly α-helical annexin repeats in the C terminus. tPA binds to the hexapeptide LCKLSL in the N terminus.

Another recognized but less known receptor for tPA is annexin A2, a member of the calcium-regulated phospholipid-binding protein which is widely expressed in various types of cells and tissues [35]. Annexin A2 was first discovered as a tPA receptor in the microglia of the central nervous system (CNS) [36]. Structurally, annexin A2 is a 36 kDa protein containing three regions: the core region, the C terminal, and the N terminal (Figure 2). The unique structure of annexin A2 allows it to dock onto cell membranes in a peripheral and reversible manner [37]. There are four highly α-helical annexin repeats within the core domain in the C terminus of annexin A2. These α-helical annexin repeats mediate the binding of annexin A2 to the cell membrane [37]. tPA has been shown to bind to the hexapeptide LCKLSL (residues 7–12) in the N terminus of annexin A2 [38]. However, unlike LRP-1, annexin A2, as a membrane-associated protein, lacks the transmembrane domain and can only dock onto the cell surface [37,39]. Therefore, it is presumable that annexin A2 requires additional co-receptors for its intracellular signal relay [37]. Our previous work has shown that integrin CD11b is one of these co-receptors, which mediates the relay of these signals triggered by the binding of tPA to annexin A2 [16]. Past studies have demonstrated the binding of tPA to annexin A2 on the surface of various cells, including macrophages, endothelial cells, and some cancer cells [16,40,41,42]. Binding of tPA to annexin A2 has been shown to play an important role in modulating ECM homeostasis, promoting cell migration, and activating microglia [36,42,43].

## 5. tPA and NF-kB Signaling in Renal Inflammation

Activation of NF-κB is a central signaling event in inflammation onset and progression. The NF-κB family consists of five members: p50, p52, p65 (RelA), RelB, and c-Rel, which form a variety of homo- and hetero-dimers [44,45]. The most common heterodimer is p50/p65, which is detectable in most cell types [46]. NF-κB family members have a common conserved Rel homology domain (RHD). The RHD is a 300-amino-acid-long region that functions as the NF-κB site for homo-/hetero-dimerization, nuclear translocation, and DNA promoter binding [46,47]. However, only p65 (RelA), RelB, and c-Rel contain the carboxy-terminal transactivation domain (TAD) which is required for transcriptional activation [46]. In an inactive state, NF-κB is sequestered in the cytoplasm by specific members of the inhibitory κB (IκB) family. The IκB proteins possess ankyrin repeat motifs interacting with the RHD of NF-κB members, which effectively inhibits and sequesters NF-κB out of the nuclei [46]. Upon activation, IκB is phosphorylated and degraded, leading to the release and nuclear translocation of p65/p50 or c-Rel/p50 (canonical activation) or RelB/p52 (non-canonical activation), and subsequently NF-κB-dependent gene transcription [44,45].

The canonical pathway is triggered by a variety of signals, such as proinflammatory cytokines and pathogen-associated molecular patterns (PAMPs), which activate cell surface receptors, including pattern-recognition receptors (PRRs), Toll-like receptors (TLR), and T-cell receptors (TCR), leading to the phosphorylation and degradation of IκB and subsequently nuclear translocation of p65/p50 or c-Rel/p50. In contrast, the non-canonical pathway responds to a more specific set of stimuli. Although there are other receptors, tumor necrosis factor (TNF) cytokines and their respective TNF receptors (TNFR) are the most well-known receptors that mediate the non-canonical NF-κB pathway. These involved TNFRs include lymphotoxin-β receptor (LTβR), B-cell-activating factor receptor (BAFFR), fibroblast growth factor-inducible factor 14 (Fn14), and others [48]. After stimulation of TNFs and pertinent receptors, NF-κB inducing kinase (NIK) is activated to initiate the non-canonical pathway, leading to nuclear translocation of RelB/p52 [44,45].

Both NF-κB and tPA have been implicated in the modulation of renal inflammation and fibrosis. NF-κB has been shown to be associated with immune-related kidney diseases, including lupus nephritis and IgA nephropathy [49,50]. NF-κB activation has also been documented in animal models of both acute and chronic kidney diseases [45,51,52]. The extent of NF-κB activation usually correlates with the severity of kidney fibrosis [53]. Additionally, NF-κB inhibition has been shown to reduce inflammatory responses and fibrosis in various CKD models, further validating the significant role of NF-κB in kidney diseases [54,55].

**Figure 3:**
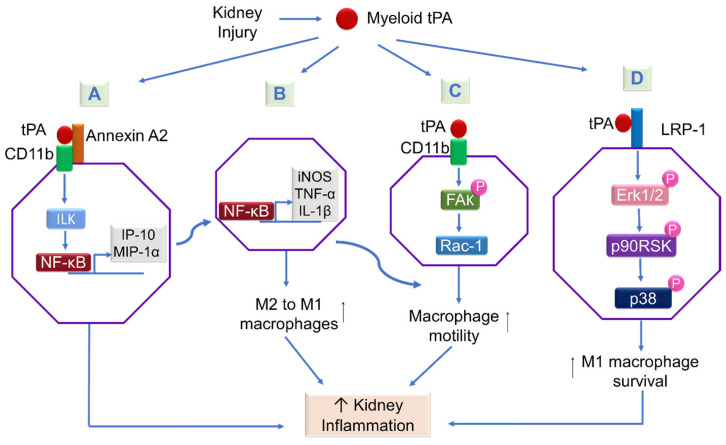
**tPA modulates macrophage function in kidney disease.** After chronic kidney injury, myeloid-derived tPA activates multiple receptor-mediated signal cascades to modulate macrophage function and promotes kidney inflammation. (**A**) tPA binds to annexin A2 and promotes aggregation of annexin A2 and CD11b leading to activation of the downstream ILK/NF-κB pathway and induction of NF-κB-dependent IP-10 and MIP-1α. (**B**) tPA promotes macrophage M2 to M1 phenotypic switch through an annexin A2/NF-κB-mediated pathway, as indicated by production of M1 chemokines such as iNOS, TNF-α, and IL-1β. (**C**) tPA activates CD11b-dependent FAK and Rac1 pathway, together with tPA-induced NF-κB, causing increased macrophage motility. (**D**) tPA binds to LRP-1 and activates downstream mediators of Erk1/2, p90RSK, and p38, promoting survival of M1 macrophages. The converging effects of the above tPA signal pathways cause substantial accumulation of M1 macrophages, sustained inflammation, and eventually tissue destruction and extensive scar formation, i.e., fibrosis, in the diseased kidney. Black upwards arrows stand for up-regulation. Blue arrows mean order of sequesntial signal transduction events.

We have hypothesized that tPA promotes renal inflammation by modulating the NF-κB pathway based on our previous findings of the concurrent induction of tPA and activation of NF-κB during the progression of CKD and the competency of tPA in regulating renal inflammatory responses [29]. We have investigated the role of tPA and NF-κB in a mouse UUO model of CKD and have found that tPA promotes kidney fibrosis and inflammation. After obstructive injury, tPA knockout mice displayed less collagen deposition, fewer CD11b-positive macrophage infiltrations, and reduced activation of NF-kB in the diseased kidneys, as indicated by lower renal p65 phosphorylation and less NF-kB-dependent chemokines. We have further demonstrated that tPA activates NF-κB signaling independent of its protease activity via the canonical pathway in macrophages by inducing IκB phosphorylation and triggering the nuclear translocation of p65/RelA, and subsequently the transcription of NF-kB-dependent chemokines, such as interferon-γ-inducible protein (IP)-10 and macrophage inflammatory protein (MIP)-1 α [16]. Intriguingly, LRP-1, as the most-studied receptor of tPA does not mediate tPA-activated NF-κB signaling, because siRNA knockdown of LRP-1 has had little effect. Instead, annexin A2 has been shown to mediate tPA’s effects. Because cell surface annexin A2 lacks the transmembrane domain and can only dock onto cell membranes in a peripheral manner [37], we have proposed that annexin A2 may function as a coreceptor of a known outside–in signal transduction pathway, such as integrin signaling. It is known that tPA binds to integrin Mac-1 (CD11b/CD18) in macrophages [23], we have investigated the possible interaction between annexin A2 and CD11b. It has been discovered that tPA promotes the aggregation of annexin A2 and CD11b in macrophages, and such interaction is also induced in the obstruction-injured kidney. Further in vitro studies have defined that tPA-induced aggregation of annexin A2 and CD11b activates its immediate downstream effector integrin-linked kinase (ILK), which, in turn, leads to the phosphorylation and degradation of IκB, release and nuclear translocation of NF-κB dimers, and subsequently DNA binding and transcription of target proinflammatory genes [16] (Figure 3A). These findings have defined a novel signaling mechanism of tPA-mediated NF-κB activation in promoting macrophage infiltration and renal inflammation. It is worth mentioning that macrophages also produce tPA, which may activate NF-κB signaling in macrophages in an autocrine manner, initiating a vicious cycle of amplification.

It should also be noted that the effect of tPA on NF-κB signaling is context-dependent and affected by many factors, such as cell types, cellular states, and organ specificities. One of the notable examples is that tPA has been shown to suppress NF-κB activation in LPS-stimulated macrophages through LRP-1-mediated miR-155 [56]. The anti-inflammatory activity of tPA is mediated through the interaction of LRP-1 and its coreceptor N-methyl-D-aspartate Receptor (NMDA-R), which is characterized as a neuron ionotropic glutamate receptor in neurons, macrophages, and Schwann cells [56,57]. LRP-1/NMDA-R-mediated effect is cellular-state-dependent [58]. Non-stimulated mouse peritoneal macrophages express very low levels of NMDA-R and are not responsive to tPA. However, after treatment with colony-stimulating factor-1 (CSF-1), activated macrophages display increased cell-surface NMDA-R and responsiveness to tPA [59]. Moreover, Zhang et al. demonstrated that tPA and LRP-1 mediate cerebral ischemia-induced NF-κB activation [18]. They found that ischemic insult-induced NF-κB activation is attenuated after LRP inhibition or tPA knockout [18]. Therefore, tPA may execute cell-type-specific biological functions by binding to different membrane receptors (LRP-1 or annexin A2) or their coreceptors, such as NMDA-R and CD11b, and initiating different intracellular signaling events to modulate NF-κB activation [16,18,56].

## 6. tPA and Macrophage Motility in Kidney Disease

Increased motility is one of the main contributing factors leading to macrophage recruitment and accumulation in the diseased kidney. tPA has been shown to be induced during the onset of macrophage and neutrophil infiltration in various injury models, indicating an important role of tPA in this process [16,17,60]. To determine whether renal endogenous tPA mediates macrophage migration and accumulation in kidney with injury, bone-marrow macrophages collected from tPA KO mice were labeled with PKH26 Red Fluorescence and intravenously injected into tPA WT and KO mice, followed by UUO injury. It was found that obstructive renal injury-induced migration of PKH26-labeled macrophages are alleviated in tPA KO mice. Additional study has demonstrated a similar redistribution of the PKH26-positive cells in the spleen, the major organ stores the homing immune cells [61], in both tPA WT and KO mice, further illuminating that local endogenous tPA is one of the driving factors of macrophage infiltration in diseased kidneys [24].

Further mechanistic in vitro studies have demonstrated that tPA modulates macrophage motility through its protease-independent cytokine function, and CD11b is indispensable to the migratory effect of tPA. It has been found that tPA activates CD11b integrin signaling by phosphorylating its downstream focal adhesion kinase (FAK) and activation of Ras-related C3 botulinum toxin substrate 1 (Rac1) [24]. FAK, an integrin signaling kinase, and Rac1, a Rho GTPase, are known to promote cell motility and spreading [62,63]. Inhibition experiments have validated that FAK acts upstream of Rac1 in mediating tPA-induced macrophage motility. Although the exact molecular detail remains unknown, FAK may regulate the complex of paxillin kinase linker (PKL/Git2) and β–pix to modulate cytoskeletal arrangement [64]. β–pix acts as an exchange factor for Cdc42 and is connected to focal adhesions through the binding of PKL/Git2 to paxillin [65]. It also serves as a scaffold to activate Rac and PAK signaling [66]. FAK induces the tyrosine phosphorylation of β–pix, leading to the recruitment and activation of Rac1 and subsequent actin cytoskeleton rearrangement and cell migration [67]. Intriguingly, NF-κB also mediates tPA-induced macrophage motility, since its inhibition attenuates the effect of tPA. Thus, it is possible that annexin A2 may act as a coreceptor of CD11b in mediating tPA-induced macrophage motility through a novel signal cascade involving the FAK/Rac-1/NF-κB pathway (Figure 3C). Of note, tPA/CD11b signaling has also been shown to modulate macrophage motility in an LPS-induced peritoneal macrophage efflux model [23].

## 7. tPA and Macrophage Phenotypic Switch in Kidney Disease

Macrophages are mixed populations of diverse cells which carry out local microenvironment-specific functions [3]. In response to various physiological or pathological cues, macrophages acquire different functional phenotypes through polarization process and differentiate into M1 or M2 subsets. M1 macrophage accumulation has been documented during the early stages of kidney injury, while M2 macrophages are more prevalent during the later stages of injury as an effort to resolve inflammation and promote repair [68,69]. Of note, M2 macrophages have also been shown to promote renal fibrosis in various animal models, including AKI-to-CKD transition and CKD models, as well as in human kidney diseases [70]. Most of these studies utilize M2 macrophage depletion or adoptive transfer to establish the pathogenic role of M2 macrophages in kidney fibrogenesis. Although the exact mechanisms remain to be elucidated, M2 macrophages are known to produce profibrotic factors, such as TGF-β1 [71]. Thus, future studies regarding the underlying mechanisms of M2 macrophage-mediated renal fibrosis are warranted.

While the mechanism underlying macrophage polarization remains not completely understood, our previous work has demonstrated that tPA preferably promotes M1 macrophage accumulation, leading to profound inflammation in an obstruction-induced CKD model, suggesting a potential role of tPA in macrophage polarization [72]. In addition, the fact that the tPA/NF-κB pathway has been shown to promote macrophage motility, a typical characteristic of M1 macrophages, further supports their role in such a process [24]. We have examined the role of tPA in macrophage polarity in a UUO model of CKD and found dramatic induction of M2 macrophage markers, such as Relm-α and Ym1, in the obstructed kidneys of tPA KO mice than those of WT mice. However, the induction of M1 chemokines, such as IL-1β, iNOS, and IP-10, in the WT mice was significantly reduced in tPA KO mice [5]. tPA-induced renal M1 macrophage accumulation and the concurrent induction of proinflammatory chemokines in the diseased kidney strongly support a role of tPA in macrophage M2-to-M1 phenotypic switch. Further in vitro experiments have demonstrated that tPA dose-dependently reduces IL-4-induced M2 makers of arginase 1 and Ym1 and reverses the M2 chemokine profile of IL-4-treated macrophages to M1 phenotype, as demonstrated by reduced IL-10 expression and up-regulation of iNOS, TNF-α, and IL-1β. Inhibition experiments have excluded the involvement of LRP-1. In contrast, annexin A2 has been shown to mediate the tPA-induced macrophage M2-to-M1 switch. Additional investigations have confirmed the essential role of NF-κB in this process. Thus, tPA induces macrophage M2-to-M1 phenotypic switch through annexin A2-mediated NF-κB pathway [5] (Figure 3B).

## 8. tPA and Macrophage Survival in Renal Injury

The number of macrophages in diseased kidneys is finely regulated by the balance among the expansion through proliferation, clearance by apoptotic death, and the recruitment of circulating monocytes [4,73,74]. Resting macrophages have a finite lifespan and presumably undergo apoptotic death locally [4]. However, differentiated macrophages, in response to pathogenic cues, display an extended life span and are resistant to apoptosis [75,76], leading to the accumulation of these cells at the sites of injury.

We have examined the role of tPA in macrophage survival in a UUO mouse model and have found that tPA-deficient mice display significantly decreased M1 macrophages, as well as associated M1 chemokines. However, the number of M2 macrophages in the diseased kidney was similar in both tPA WT and KO mice. There were more apoptotic M1 macrophages in the injured kidneys from tPA KO mice than those from WT mice. In vitro experiments have shown that tPA-treated macrophages are resistant to apoptosis induced by at least two types of apoptosis-inducing agents and promote the survival of resting macrophages and LPS- or IFN-γ-induced M1, but not IL-4-induced M2, macrophages through tPA protease-independent cytokine function. Inhibition experiments have demonstrated that LRP-1 mediates the cytoprotective effects of tPA through a novel signal cascade involving Erk1/2, p90RSK, and p38, which results in substantial accumulation of M1 macrophages in the injured kidney [72] (Figure 3D). Notably, p90RSK is a serine/threonine kinase that phosphorylates a variety of downstream substrates, including IκB [77,78,79]. Thus, tPA/LRP-1 signaling may also indirectly activate the NF-κB pathway through p90RSK-mediated IκB phosphorylation and further amplify NF-κB-dependent proinflammatory signaling, such as the macrophage M2-to-M1 phenotypic switch, resulting in a vicious loop of amplification and excessive kidney inflammation. Together, it is clear that tPA promotes M1 macrophage accumulation in the diseased kidney in response to chronic injury cues, not only by enhancing the survival of these macrophages, but also through inducing macrophage M2-to-M1 phenotypic switch. Intriguingly, although tPA promotes M2 transition to M1 macrophages, the renal M2 macrophage population is largely unchanged in both tPA WT and KO mice after obstructive injury, suggesting a tPA-independent mechanism in modulating the number of M2 macrophages in response to chronic injury.

## 9. Conclusions

As summarized in Figure 3, in response to chronic kidney injury, interstitial induction of myeloid-derive tPA modulates macrophage function through activating multiple receptor-mediated signaling pathways: (1) binds to annexin A2 and activates the CD11b signaling cascade of ILK and NF-κB, leading to proinflammatory M1 chemokine expression and renal inflammation; (2) activates the CD11b-dependent FAK and Rac1 pathway, as well as NF-κB, to promote macrophage migration and infiltration into damaged kidney; (3) activates the annexin A2/NF-κB pathway to induce renal macrophage M2-to-M1 phenotypic switch; and (4) binds to LRP-1 and activates its downstream signaling cascade of Erk1/2, p90RSK, and p38 to promote the survival of M1 macrophages in the diseased kidney. The synergetic effect of these signaling events will substantially enhance renal accumulation of M1 macrophages, leading to excessive renal inflammation. Thus, it is clear that tPA modulates diverse macrophage functions through various receptor-mediated signal pathways and plays an important role in the pathogenesis and progression of kidney inflammation and fibrosis. Notably, many of these effects are context-dependent, and tPA may have opposite effects in different cellular states and organ systems. While we have garnered significant understanding of regulatory mechanisms of tPA in macrophages *per se*, little is known about its role in intercellular communication between macrophages and other kidney cells. Future studies are warranted to investigate the role and underlying mechanisms of tPA signaling in the interactions between macrophages and renal parenchymal cells, such as interstitial fibroblasts and tubular epithelial cells, during AKI-to-CKD transition and CKD progression, and develop therapeutic strategies targeting pathogenic tPA signaling in kidney diseases.

## Data Availability

Not applicable.
